# Method for the identification of pharmacological intervention for the disruption of fear memory in PTSD-rat model

**DOI:** 10.1016/j.mex.2020.101059

**Published:** 2020-09-09

**Authors:** Saida Haider, Zehra Batool, Sahar Rafiq

**Affiliations:** a Neurochemistry and Biochemical Neuropharmacology Research Unit, Department of Biochemistry, University of Karachi, Karachi 75270, Pakistan; b Dr. Panjwani Center for Molecular Medicine and Drug Research International Center for Chemical and Biological Sciences, University of Karachi, Karachi, Pakistan

**Keywords:** PTSD-model, Rats, Consolidation, Reconsolidation, Extinction

## Abstract

A large portion of the human population is exposed to traumatic events once in their lifetime, 10% of which may undergo post-traumatic stress disorder (PTSD). It is a mental condition triggered by a traumatic event resulting in severe anxiety disorder which may severely affect the daily routine life of the individual. The patient expresses the aversive memory by recalling any fear event related to the traumatic experience. The disruption of fear memory related to fear event is one of the best approaches to treat PTSD. In this regard, pharmacological interventions provide a possible way to erase or lessen the fear memory of the traumatic event. The screening and identification of drugs is one of the crucial steps to introduce new potent drugs in preclinical setup. Pavlovian fear conditioning is the well known experimental protocol to study fear memory. In this article, we are presenting a detailed method of Pavlovian fear conditioning which we have optimized in our lab for the identification of drugs having the potential to disrupt fear memory in the PTSD-rat model. In this protocol, various stages of memory formation including consolidation, reconsolidation, and extinction have been targeted to study the effect of a particular drug.

• The protocol provides step by step procedure to study the effects of known or putative drugs in an animal model of PTSD.
• The method also explains the separate protocols to target specific stages of memory so that one can identify the effects of drugs on a particular phase of remote or recent memory formation.

## Graphical abstract

**Figure:**
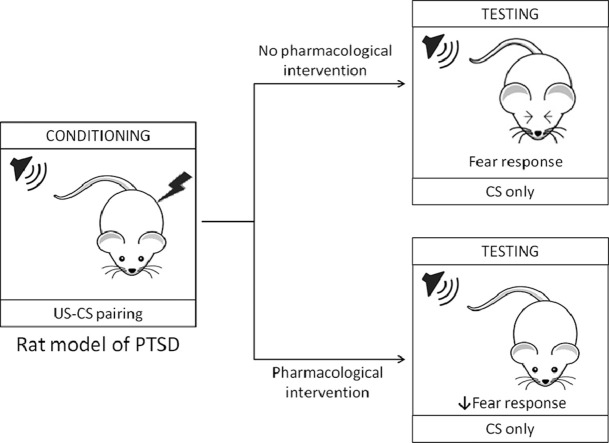

Specifications table

**Table:** 

Subject Area	Neuroscience
More specific subject area	Neuropharmacology
Method name	Pavlovian fear conditioning
Name and reference of original method	M.S. Monsey, D.M. Gerhard, L.M. Boyle, M.A. Briones, M. Seligsohn, G.E. Schafe, A diet enriched with curcumin impairs newly acquired and reactivated fear memories, Neuropsychopharmacology 40(5) (2015) 1278–1288.
Resource availability	Rats, aparatus, training chamber, testing chamber, Ethanol 70%, Stop watch.
Trial registration: Ethics:	The procedures were performed in accordance to the policy of institutional Advanced Studies and Research Board (ASRB/47821/*Sc*) and executed in line with National Institute of Health Guide for Care and Use of Laboratory Animals (Publication No. 85-23, revised 2011).

## Method details

Post-traumatic stress disorder (PTSD) is considered as a traumatic or stressor related disorder and represents one of the major burden in society at large. The Pavlovian fear conditioning methodology has been used frequently to examine the acquisition and reduction of fear response in an animal model [14]. It is one of the widely and commonly used paradigms to study PTSD-associated disorder models to translate these findings to human populations [6]. In recent years research has focused on using animal models to develop and identify the pharmacological agents that can disrupt the fear memory. The current method aims to describe how the animal models of PTSD can be helpful to identify pharmacological intervention which can disrupt the fear memory process at different stages of memory formation. We primarily targeted fear memory by interfering at different stages like consolidation, reconsolidation, and extinction phase in an animal model of PTSD. Before discussing the method to identify pharmacological interventions, we first briefly overviewed the commonly used rodent models of fear and anxiety such as PTSD.

Ivan Pavlov was a scientist who gave an initial idea of classical conditioning in which the subject learns that conditioned stimulus (CS) predicts the occurrence of the unconditioned stimulus (US) [19]. One form of Pavlovian conditioning that has received considerable attention in the last 10 years is fear conditioning [12]. The Pavlovian studies were used by other scientists to design the classical fear conditioning paradigm, therefore, called Pavlovian fear conditioning which is widely used to study emotional memories [1,5]. Fear learning is an adaptive and normal process that allows an individual to acquire defensive behavior for the survival in response to environmental threat or cues. During Pavlovian fear conditioning, the neutral or CS such as tone or light is paired with the aversive or US such as electric shock to the feet or air puff to the eyes. When subject is exposed to CS only, it results in conditioned response such as freezing due to the association of CS with the US. Freezing is defined as the cessation of all movement except movement related to respiration and it indicates the fear response in an animal. Once the association of CS-US develops in an animal, memory of this fear association can be later tested by presenting the CS in the absence of the US. This form of Pavlovian conditioning is very robust and it can be induced with a single pairing of CS-US and has been shown to operate in a wide variety of species, from flies to humans [11].

The mechanism of inhibition of fear conditioning using pharmacological intervention has become a subject of great interest. The pharmacological intervention is considered as an important tool to facilitate the impairment of fear memory. Therefore, the identification of a therapeutic drug that can impair consolidation and reconsolidation of fear memory or facilitate the extinction of fear memory is necessary for the course of treatment of PTSD and related anxiety disorders in the clinical setup. Moreover, the screening of putative pharmacotherapies to target the impairment of recent or remote memory also requires a specific protocol of Pavlovian fear conditioning. In the following sections, we are explaining the protocols for testing the effectiveness of suggested pharmacological interventions to impair specific phase of fear memory in a rat model of PTSD.

## Standard method of Pavlovian fear conditioning

### Habituation

Animal is placed in a training chamber having a wire grid in transparent activity box for 15 min to avoid any psychological stress related to the environment. The locomotor activity during habituation is an indication which ensures the researcher that animal is free from any fear or stress.

### Training

After 24 h of habituation, a training session is conducted in the training chamber cleaned with ethanol. When an animal is placed in the chamber, freezing behavior is noted for 3 min which is taken as baseline freezing. During this time, the animal should not exhibit freezing behavior. This is used as a measure of non-specific freezing to the context. After 3 min baseline period, a tone (auditory) cue is then presented, generally at a level of 70–80 dB for 15–30 s. A mild foot shock is administered during the last 2 s of the tone presentation and co-terminates with the tone. The foot shock (0.17–0.8 mA) is presented for 1–2 s. After the shock presentation, an inter-trial interval for about 60–210 s precedes a second identical trial. In the standard paradigm, rodents are trained in fear-conditioning chambers and receive 1–10 tone-shock pairings for the course of 5–10 min. Following the final shock presentation the animal should be removed from the activity box in a 30–60 s time period after the last trial [4]. The chamber is then cleaned and dried before starting the session for the next animal.

### Testing

Testing of fear memory is conducted in the testing chamber sprayed with distinct odor, to change the odor of context. Testing session is conducted by the presentation of CS in the absence of the US. During the testing phase, the freezing behavior is noted for 3 min before the presentation of tones and taken as baseline freezing. Latency to freeze is the time at which the animal starts to freeze. After 3 min baseline period, CS is presented consisting of the same intensity as applied during training phase. The freezing posture during the presentation of CS is taken as freezing during tone. After the last trial animal is left in the chamber for an additional 1 min to reduce the hyperactivity of animals. The chamber is then cleaned and dried before starting the next session. This overall experimental scheme is often modified depending on the needs of the experimenters.

## Drug application for targeting a particular phase of fear memory

### Materials

Activity transparent box (26 × 26 × 26 cm)

Iron grid floor

Plastic floor

Wood cedar chips

70% ethanol

Pepper mint spray

Electric circuit (0.5 mA)

Stop watches

Camera

Sound of bell (75 dB)

Sand paper for cleaning iron grids

Tissue paper or towel for cleaning purpose

Leather gloves for protection as rats get hyper after conditioning phase

**Parameters for observation:** Observe freezing posture as it is an index of fear memory.

Latency to freeze during baseline (freezing time for the onset of freezing posture)

Baseline freezing (freezing time before tone)

Latency to freeze during tone (freezing time for the onset of freezing posture during tone)

Freezing during tone (freezing time during tone)

## Consolidation

Converging studies on the consolidation process have used associative learning tasks like Pavlovian fear conditioning [21]. To substantiate memory consolidation, memory performance is often assessed 24 h after encoding. Retention of the fear memory is inferred from the behavioral conditioned response (CR).

**Apparatus**: Plastic chambers having the dimensions of 26 × 26 × 26 cm with iron-grid floor present in a sound-attenuating room. The electric circuit should be connected with iron-grids.

**Chambers:** Two chambers are required

1. Training chamber is a brightly illuminated and transparent chamber equipped with a grid-iron floor.
2. Testing chamber is a transparent chamber and having a black plastic flat floor.

**Sessions**: Three sessions including habituation, training, and testing.

**Drug administration:** Immediately following training session.

**Method for observing freezing posture**: It is done manually by using stop watch.

**Cleaning:** Clean with 70% ethanol and use paper towel to clean urine and feces.

**Odor:** Peppermint spray to change the context during the testing phase.

## Method

**Fig. 1:**
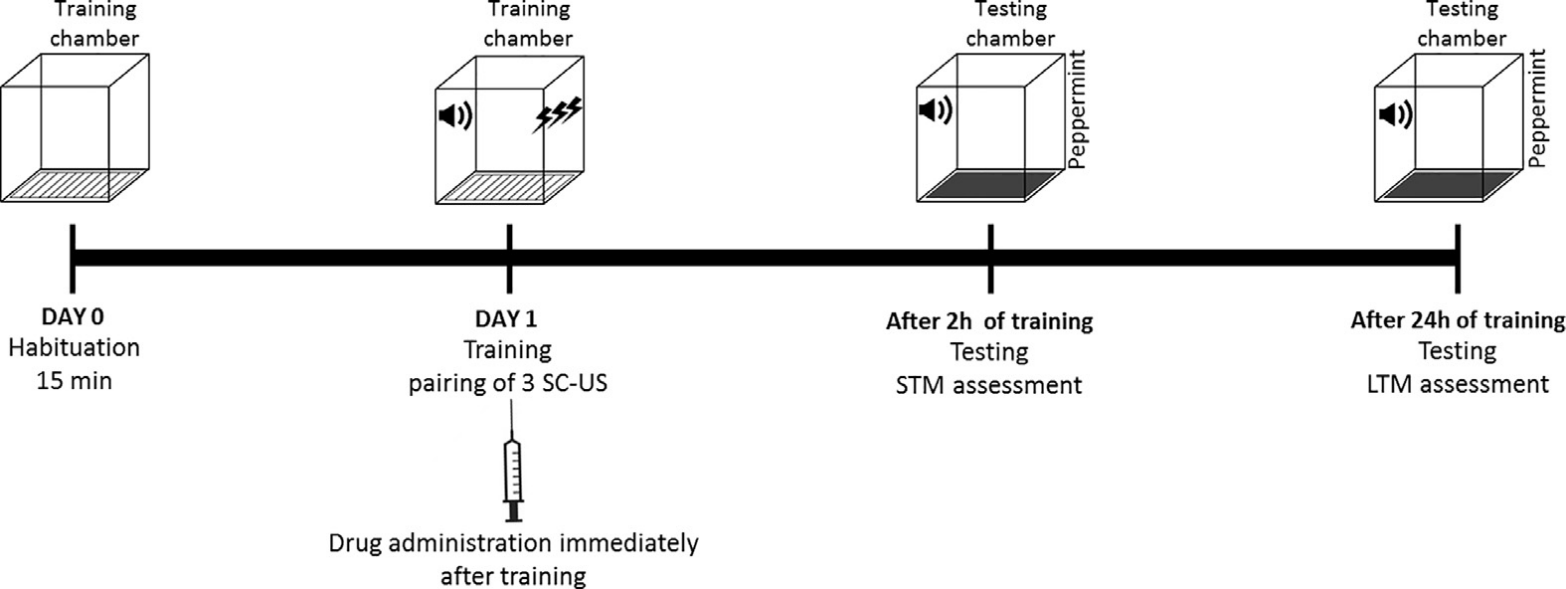
Protocol timeline for targeting consolidation phase of fear memory in animal model of PTSD.

Initially the animal is habituated in training chamber for 15 min. Following day, training of an animal is conducted in training chamber by presenting 3 trials of CS-US consisting of 20 s, 5 kHz, 75 dB tone (CS) which is co-terminated with a brief foot shock of 0.5 mA for 1 s (US). For the disruption of consolidation of fear memory, the drug is administered immediately after training so that memory traces related to that traumatic experience would not get into a stable state. Short-term memory (STM) is analyzed 2 h after training by presenting three CS and after 24 h, long term memory (LTM) is assessed by presenting 10 CS (Fig. 1). All new memories undergo a consolidation process and interrupting this process prevents the transition from STM to LTM [16]. The application of pharmacological intervention during consolidation process may interrupt the transition of STM to LTM. Due to interferences, memory traces are unable to attain stabilized form and remain in a labile state that is responsible for the impairment of consolidation of fear memory. The use of pharmacological agent is known to disrupt various processes such as synaptic alteration, intracellular cascades, and, protein synthesis that are responsible for consolidation of the memory.

## Reconsolidation of recent fear memory

Reconsolidation is a dynamic process by which information is integrated or removed in existing memory traces [2]. For the disruption of reconsolidation of fear memory, it should be retrieved first, which is done by “retrieval trial” (giving a reminder of the original experience) [9]. New insights suggested that pharmacological interventions are suited to modify or remove fear memory by targeting the reconsolidation process whether it is a newly formed or an older formed fear memory [22].

**Apparatus:** Plastic chamber having the of dimensions 26 × 26 × 26 cm with iron-grid floor present in a sound-attenuating compartment. The electric circuit connected with iron-grids.

**Chambers:** Two chambers are required

1. Training chamber is a brightly illuminated and transparent chamber equipped with a grid-iron floor.
2. Testing chamber is a transparent chamber having black plastic flat floor.

**Sessions**: Four sessions; habituation, training, reactivation, and testing.

**Drug administration:** Immediately following reactivation session.

**Method for observing freezing posture**: It is done manually by using stop watch.

**Cleaning:** Clean with 75% ethanol and use paper towel to clean urine and feces.

**Odor:** Peppermint spray during testing to change the context

## Method

**Fig. 2:**
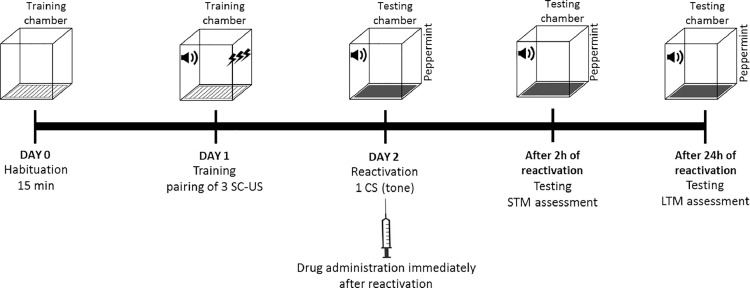
Protocol timeline for targeting reconsolidation phase of recent fear memory in animal model of PTSD.

Habituation is carried out in training chamber for 15 min. Following day, training of an animal is conducted in training chamber by presenting 3 trials of CS-US. Reactivation of fear memory is conducted 24 h following the training session by presenting a single CS only in the testing chamber. For the disruption of reconsolidation of recent fear memory, drug is administered immediately after reactivation of memory. STM is analyzed 2 h after reactivation by presenting three CS and LTM is assessed after 24 h by presenting 10 CS (Fig. 2). Recalling or re-exposure of traumatic event may return the stabilized fear memory traces to be in a labile or fragile state again and provide a new window of opportunity to modify the fear memory by pharmacological intervention. It has been suggested that application of interventions during reconsolidation may modify the original fear memory and thus prevent the fear response.

## Reconsolidation of remote fear memory

The age of memory is an important factor which shows the stability of retrieved memory. Recent memories are more susceptible to disruption while older memories are difficult to disrupt [23]. New insights suggested that pharmacological interventions are suited to modify or remove fear memory by targeting the reconsolidation process whether it is a newly formed or an older formed fear memory [22].

**Apparatus:** Plastic chamber having the dimensions of 26 × 26 × 26 cm with iron-grid floor contained in a sound-attenuating compartment. Electric circuit connected with iron-grids.

**Chambers:** Two chambers are required

1. Training chamber is a brightly illuminated and transparent chamber equipped with a grid-iron floor.
2. Testing chamber is a transparent chamber and having black plastic flat floor

**Sessions**: Four sessions; habituation, training, reactivation, and testing.

**Drug administration:** After reactivation session.

**Method for observing freezing posture**: It is done manually by using stop watch.

**Cleaning:** Clean with 75% ethanol and use paper towel to clean urine and feces.

**Odor:** Peppermint spray for changing the context during test session.

**Method**

**Fig. 3:**
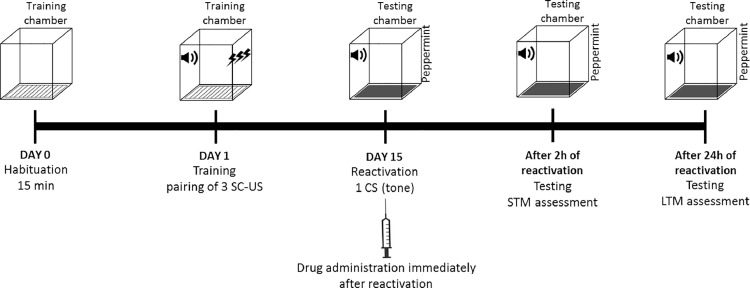
Protocol timeline for targeting reconsolidation phase of remote fear memory in animal model of PTSD.

Habituation is conducted in a training chamber for 15 min. Following habituation, training of an animal is conducted in the training chamber by presenting 3 trials of CS-US. For the disruption of re-consolidation of remote fear memory, reactivation is carried out after 2 weeks (14 days) of training session by presenting a single CS. Reactivation phase is then followed by drug administration. STM is analyzed 2 h after reactivation by presenting three CS, and LTM is assessed after 24 h by presenting 10 CS (Fig. 3). Pharmacological interceding can modify remote fear memories which are considered difficult to disrupt [23]. By targeting the reconsolidation process, retrieved memories are re-stabilised even though they may be decades old [16]. Pharmacological intervention of reconsolidation might be of great clinical importance for the treatment of psychiatric disorder.

## Extinction

Extinction is an essential behavioral technique that occurs after repeated exposure to CS in the absence of US. This induces the formation of new learning rather than the erasure of previously conditioned fear association, causing a reduced fear expression [25]. Treatment of PTSD by extinction process suggests the modulation of fear memory [18] but 23–27% of patients that undergo such exposure-based therapies experience a relapse [3,8]. So here the pharmacological manipulation comes as an adjuvant to play its role with extinction process.

**Apparatus**: Plastic chamber having the dimensions of 26 × 26 × 26 cm with iron-grid floor contained in a sound-attenuating compartment. The electric circuit should be connected with iron-grids.

**Chambers:** Two chambers are required

1. Training chamber is a brightly illuminated and transparent chamber equipped with a grid-iron floor.
2. Testing chamber is a transparent and having black plastic flat floor

**Sessions**: Three sessions; habituation, training, extinction, and testing.

**Drug administration:** Immediately following training session.

**Method for observing freezing posture**: It is done manually by using stop watch.

**Cleaning:** Clean with 70% ethanol and use paper towel to clean urine and feces.

**Odor:** Peppermint spray for changing the context during testing

**Method**

**Fig. 4:**
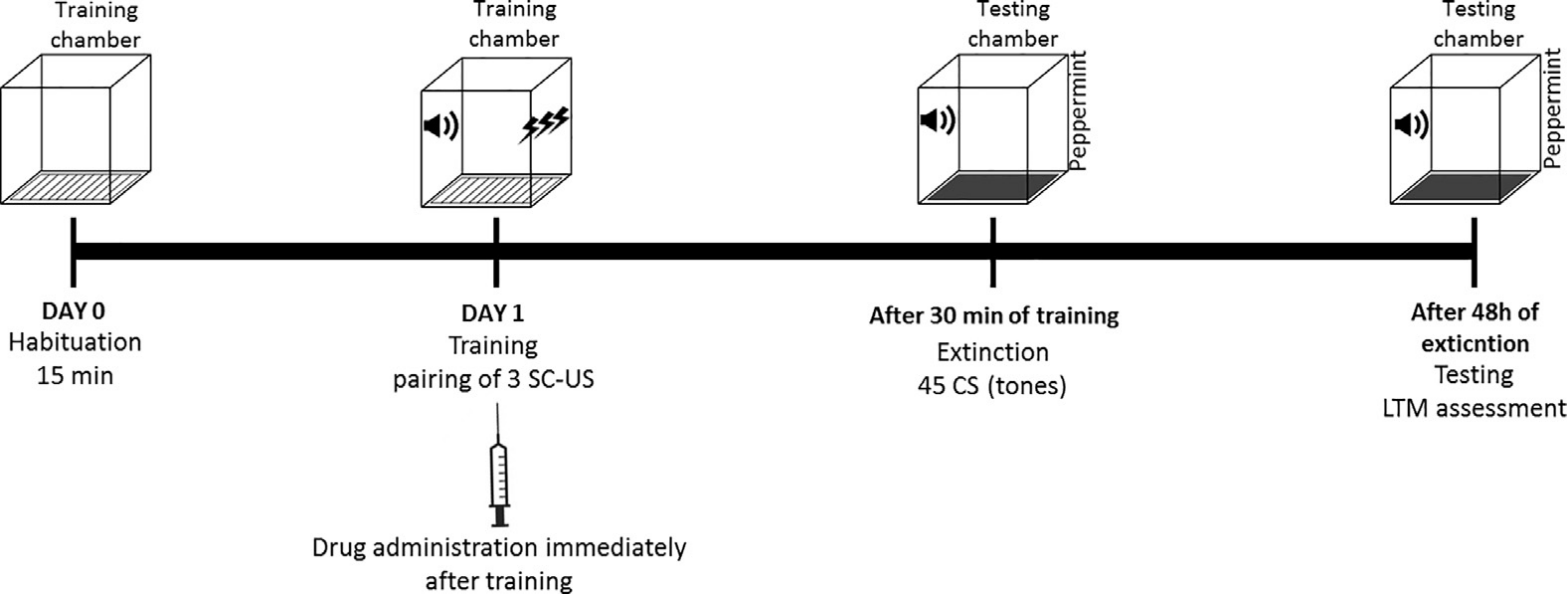
Protocol timeline for pharmacological intervention along with extinction procedure in rat model of PTSD.

Animal is habituated in training chamber for 15 min. Following day, training of an animal is conducted in the training chamber by presenting 3 trials of CS-US. Immediately after the presentation of last tone an appropriate treatment is administered. Extinction session is conducted after 30 min of training in testing chamber by presenting 45 deliveries of CS. Following 48 h of extinction of fear memory, retention of memory is tested by presenting 10 CS in the testing chamber (Fig. 4). Application of intervention after training may interfere with the fixation of fear memory trace. Moreover, the occurrence of extinction process by giving multiple trials of CS makes a new memory of safety sense of CS which was previously an indicator of danger and later attains a stable form. These dominant extinct traces of memory suppress the conditioned response in the rat model. It has been proposed that interventions applied along with extinction process may modify the original fear memory and thus prevent the spontaneous recovery and reinstatement of the fear response.

A complication of interpreting studies of reconsolidation is that extinction can occur under similar conditions after memory retrieval. Extinction occurs after repeated presentations of the CS resulting in a gradual decrement of the conditioned response (freezing). Extinction tends to weaken the original memory by the formation of new memory [24]. Length of retrieval session, number of CS presentation, and strength of memory are identified as boundary conditions which differentiate between reconsolidation and extinction phases. Behaviorally there is a difference in the duration of CS exposure, reconsolidation usually requires limited exposures of the CS whereas extinction requires multiple exposures of CS [10]. Merlo and colleagues demonstrated the effect of an increased number of CS presentation during the retrieval window. They presented 1 CS to 10 CS in order to examine the dominant process (reconsolidation or extinction) following reactivation of memory. They showed that memory reactivation by presenting a single or few CS (fewer than four) will induce the labilization and subsequent reconsolidation of the original fear memory and further presentations of the CS (e.g., four presentations) cancels the lability of the original memory and prevents reconsolidation, but fails to engage extinction. Finally, a greater number of CS presentations (7–10 CS presentations) gradually lead to extinction [13]. It has been known that extinction is a process that involves new learning and inhibits the expression of previously acquired memories. During extinction training, a CS is repeatedly presented without the US, resulting in dissociation of CS with fear response [15]. A critical parameter that determines whether pharmacological treatment will impair reconsolidation or extinction is the length of the memory reactivation phase and extinction training sessions [7,^20^,23]. When the session is brief, reconsolidation process is dominant, whereas longer session of reactivation induces extinction mechanism [17]. Likewise, our presented protocol also showed that to target the reconsolidation process of memory only a single CS exposure (brief session) is used to reactivate the memory whereas extinction training session consisted of 45 CS exposures (longer session) following learning of fear memory. However, the testing phase for both reactivation and extinction consists of 10 CS during which the effectiveness of drug administration is monitored to impair the fear memory as compared to control animals. If the drug is effective to impair the fear memory then it would show decreased freezing response as compared to control animals.

## Consideration regarding experiment

1. Transfer the animals (rats) to the experimental room from laboratory room 1 h before starting the experiment to acclimatize the animals to the environment of the experimental room. The export of animal minimizes all types of risks of sound interruption and jerking of cages for avoidance of any emotional changes in rodents.
2. During this 1 h, set all of your apparatus and experimental necessities.
3. Before the initiation of the experiment, iron-grids of the training chamber should be rubbed with sand paper so that electric current easily passes to feet of rats without any doubt.
4. Use dim light in the experimental room during experiments because bright light distracts the activity of rats.
5. Turn on the electric circuit of the apparatus.
6. Make videos of each session as it will be helpful for further re-evaluation of data.
7. Always clean the floor and walls of the chamber before placing an animal in the chamber.
8. Odor is one of the most important factor regarding the change of environment or context because animal relates the visual cues with sensory cues. For instance, alcohol can be used as an odor for the training chamber and peppermint odor can be used for the testing chamber.
9. Always place rat next to the wall of the chamber as by placing in the center can put an animal in a stressful situation.
10. As it is a fear inducing behavior, after experiencing electric shock animal will become hyperactive, so handle the animal gently when transferring from chamber to cage.
11. Also clean the chamber floor and walls after each session.

## Declaration of Competing Interest

The authors declare that they have no known competing financial interests or personal relationships that could have appeared to influence the work reported in this paper.
